# Influence of Caretakers’ Health Literacy on Delays to Traumatic Brain Injury Care in Uganda

**DOI:** 10.5334/aogh.2978

**Published:** 2020-10-06

**Authors:** Chinemerem Nwosu, Charis A. Spears, Charles Pate, Deborah T. Gold, Gary Bennett, Michael Haglund, Anthony Fuller

**Affiliations:** 1 Duke University School of Medicine, US; 2 Duke Global Health Institute, US; 3 Duke University Medical Center, Departments of Psychiatry and Behavioral Sciences, Sociology, Psychology and Neuroscience, US; 4 Duke University, Department of Psychology and Neuroscience, US; 5 Duke Global Neurosurgery and Neurology, US

## Abstract

**Background:**

Traumatic brain injury (TBI) is a life-altering condition, and delays to care can significantly impact outcomes. In Uganda, where nurse shortages are prevalent, patients’ family members are the primary caretakers of these patients and play an important role in ensuring patients’ access to timely care. However, caretakers often have little or no knowledge of appropriate patient care. Caretakers’ ability to navigate the healthcare system and find and use health information to support their patients can impact delays in seeking, reaching, and receiving care.

**Objectives:**

This study seeks to determine the factors that impact TBI patient caretakers’ health literacy and examine how these factors influence delays in care.

**Methods:**

This study was carried out in the Mulago National Referral Hospital neurosurgical ward, where 27 adult caretakers were interviewed using semi-structured, in-depth, qualitative interviews. “The Three Delay Framework” was utilized to understand participants’ experiences in seeking, reaching, and receiving care for TBI patients. Thematic content analysis and manual coding was used to analyze interview transcripts and identify overarching themes in participant responses.

**Findings:**

The main health literacy themes identified were Extrinsic, Intrinsic and Health System Factors. Nine sub-themes were identified: Government Support, Community Support, Financial Burdens, Lack of Medical Resources, Access to Health Information, Physician Support, Emotional Challenges, Navigational Skills, and Understanding of Health Information. These components were found to influence the delays to care to varying degrees. Financial Burdens, Government Support, Emotional Challenges, Physician Support and Lack of Medical Resources were recurring factors across the three delays.

**Conclusion:**

The health literacy factors identified in this study influence caretakers’ functional health literacy and delays to care in a co-dependent manner. A better understanding of how these factors impact patient outcomes is necessary for the development of interventions targeted at improving a caretaker’s ability to maneuver the healthcare system and support patients in resource-poor settings.

## Background

The global incidence of traumatic brain injury (TBI) is disproportionately concentrated in low- and middle-income countries (LMICs) [1] and is associated with substantial morbidity and mortality. In addition to its detrimental effects on health, TBI presents a financial burden to families, healthcare systems, and economies through lost productivity and high healthcare costs [2]. In hospitals across many African countries, family members are the primary caretakers of patients during and after their hospital stays [3,4]. As of 2011, there is a 42% shortage of nurses within the public health sector in Uganda and a 10% shortage at the largest public hospital in the country, Mulago National Referral Hospital (MNRH); this has severely affected health delivery services [5]. With the scarcity of health workers, caretakers with little to no training in patient care are entrusted with relatively high-level tasks. Depending on the diagnosis and severity of illness, caregivers may engage in various care tasks such as wound care, medication and symptom management, transportation, and the provision of emotional support [6]. These tasks are particularly relevant in a neurosurgical ward, where many TBI patients are immobile and unable to speak or comprehend instructions. Caretakers play a significant role in the care and support of patients with chronic illnesses and seek health information to facilitate this role [7]. They are also an essential source of health information for patients and assist in decision-making throughout the care continuum.

Three types of health literacy have been defined based on their application in the real world: functional, interactive, and critical [8]. Functional health literacy encompasses the basic skills needed to understand health information and use it to navigate the health system [9] and differs from interactive and critical health literacy, which involve assessing more advanced cognitive and literacy skills. Functional health literacy is, therefore, critical to determine a broad understanding of health education needs. Key components of caregiver health literacy have been previously identified, including support systems, managing the challenges of caregiving, the relationship between care providers and care recipients, psychosocial support, self-care, and processing health information [7]. However, previous studies show that the impact of health literacy is usually evaluated based on the patient’s understanding, while that of caretakers of adult patients often goes underrepresented [10], revealing a significant gap in the literature.

Caretakers’ functional health literacy is likely to play a role in delays in TBI care due to their key position in making relevant health decisions on behalf of their patients. The inability to seek care immediately for patients—who may not be able to care for themselves—can significantly impact health outcomes. Delays to TBI care account for 50% of mortality within the first two hours of the incident [11] and have been previously shown to impact mortality in this population [12]. Patients with TBI have poorer health outcomes when access to post-acute emergency care is delayed, even with abundant medical resources and adequate transportation systems [13]. Consequently, these delays are likely to be exacerbated in resource-poor settings. The Lancet Global Commission on Surgery adapted the “three delays” framework, created by Thaddeus and Maine, which describes the barriers within the healthcare systems during seeking, reaching, and receiving care in LMICs that prevent patients from obtaining surgical care [14].

Investigating the factors that influence a caretaker’s ability to navigate the healthcare system and delays to care for TBI patients is fundamental to designing effective interventions tailored towards addressing health literacy needs, particularly at the initial stages of the healthcare continuum. In this study, we evaluate the factors that affect the functional health literacy of caretakers of TBI patients in a tertiary care hospital in Uganda and how they influence delays in seeking, reaching, and receiving care. These data will provide a better understanding of the extent to which caregivers can comprehend health information, which is critical for improved patient outcomes.

## Methods

### Setting

Mulago National Referral Hospital (MNRH) in Kampala, Uganda, was the site for this study and is the main referral hospital for the entire country, with a catchment area of 1.5 million and the highest level of medical and surgical expertise [15]. At the time of this study, the MNRH neurosurgery ward was staffed by four neurosurgeons, six neurosurgical residents, and five ward-specific nurses and treated an average of 50 patients daily.

### Participants

In this qualitative study, data were collected via a convenience sampling of adult caretakers of TBI patients at MNRH. Participants were interviewed for 30–60 minutes and were offered snacks after the interview as compensation for their time. In order to participate in the study, the participants must have met the following criteria: (1) serving as a caretaker for a TBI patient assigned to the neurosurgical ward at MNRH, (2) proficient in a language understood by both the interviewer and the study participant, and (3) able to coherently answer all interview questions.

### Qualitative Interview

In the semi-structured, in-depth interviews, participants were asked about their experiences and perceptions when seeking, reaching, and receiving care. Reporting of this study followed the Consolidated Criteria for Reporting Qualitative Research (COREQ) guidelines, which include a 32-item checklist for reporting the important aspects of a qualitative research study [16].

The in-depth interviews were divided into three sections. The first section included information on the purpose of the study and the structure of the interview. The second section provided the definitions of delays and barriers to care in the context of the interview. The third section posed questions about the three delays to care. Each of the delays was subdivided into a description of the delay, the time period in question, and a question regarding the participant’s proposed solutions for managing delays to care.

### Data Analysis

Each interview was transcribed verbatim by two research assistants, and inaudible or crosstalk events were noted in the transcription. Differences in the two transcripts were discussed by researchers until resolved. The data were analyzed inductively using content analysis on NVivo 12.1 software (QSR International, Melbourne, Australia). For data analysis, the researcher first read through the transcripts and identified broad themes related to caretaker functional health literacy. From these, three main themes were identified from perceptions and topics that were recurrent in various interviews. Within each theme, three sub-themes were identified for perceptions and topics related to the main theme.

Manual coding categorized the caretaker functional health literacy themes and sub-themes under the three delays model. For each of the three delays, the transcript was analyzed for sub-themes that were recurring in the interviews and then categorized according to the main health literacy theme with which it was most consistent. During coding, a tally system was used to denote the number of participants who mentioned a specific sub-theme in their interview; this was continued until saturation was reached (i.e., where no new themes were evident).

### Ethics and Consent

The ethical review boards at Duke University (Pro00069190) and the Mulago Hospital Research and Ethics Committee (MREC 1177) approved all study procedures. Before each interview began, each participant provided informed consent, and no information provided in the interviews could be connected to specific care providers.

## Study Findings

**Figure 1:**
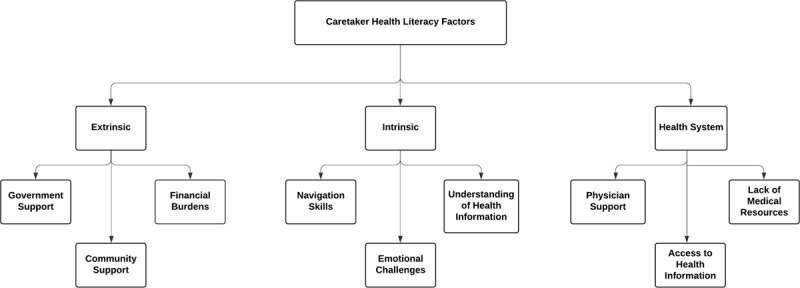
Caretaker health literacy main themes and sub-themes. Overview of all caretaker health literacy factors and their overarching themes. These were analyzed within each of the three delays to care.

In this study, 27 participant interviews generated 108 pages of transcribed interviews. After analyzing the data, all the sub-themes were grouped under three main themes, as shown in Figure 1: *Intrinsic Factors, Extrinsic Factors*, and *Health System Factors*. ***Intrinsic Factors*** include the health literacy elements inherent to the caretakers that impact their approach to caregiving and their ability to process health information. ***Extrinsic Factors*** include the health literacy elements that influence caretakers’ capability to find, understand, and use health information to care for patients. ***Health System Factors*** include the factors related to hospitals and clinics in Uganda that impact caretakers’ health decision-making and delays to care.

**Figure 2:**
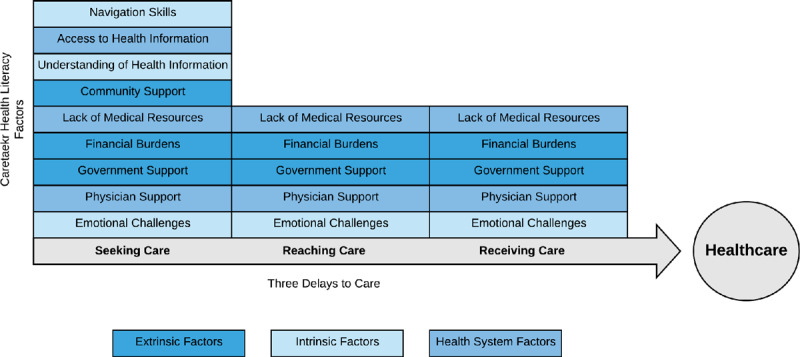
Conceptual framework of the three delays to care and caretaker health literacy factors.

The recurrent sub-themes were then grouped into the Three Delays to Care categories: Seeking Care, Reaching care, and Receiving Care, as presented in Figure 2. In the Seeking Care delay category, the recurring sub-themes included: *Community Support, Navigation Skills, Access to Information, Emotional Challenges, Understanding Health Information*, and *Lack of Medical resources*. In the Reaching Care delay category, the main sub-themes were *Lack of Medical Resources* and *Physician Support*. In the Receiving Care delay category, recurring sub-themes were *Emotional Challenges, Financial Burden, Government Support, Lack of Medical Resources*, and *Physician Support.* As demonstrated in Table 2, *Financial Burden, Government Support, Lack of Medical Resources, Emotional Support*, and *Physician Support* were the main caretaker health literacy factors found in all delay categories.

### Factors Influencing Delays to Seeking Care

*Financial Burdens* were cited by caretakers as a cause for the majority of the delays when seeking care (Table 1). Some concerns were the cost of public transportation and the high cost of past medical expenses, which led to deterrence against seeking care, as shown in the quote below:

“There was no transport like an ambulance to bring my brother here, and getting money for public transport was another barrier which delayed us until we reached here after three days.”

**Table 1:** Health literacy themes and sub-themes related to seeking care.

Main Theme	Sub-Theme	Key Examples	% of Caretakers Identifying Sub-Theme*

Extrinsic	Financial Burdens	Buying fuel to transport patient in private cars or ambulances	33.3
		Cost of public transportation	
		Initial cost of care in clinics and hospitals	
	Community Support	Relying on community members to seek care on behalf of caretakers	25.9
		Assistance with fundraising for transport	
	Government Support	Providing medical services such as ambulances and medical tests at lower costs	18.5
		Bettering roads and the public transportation system	
Intrinsic	Emotional Challenges	Feeling overwhelmed, scared, tired, and/or confused	25.9
	Navigation Skills	Looking for the hospitals or clinics after the injury	18.5
		Many referrals from clinic to clinic	
	Understanding of Health Information	Patient and caretaker did not know the severity of illness	0.04
Health Systems	Access to Information	Lack of knowledge on the injury or patient’s condition	25.9
		Uncertainty regarding where to get help	
		Lack of medical personnel to assist and provide information	
	Lack of Medical Resources	Poor access to medical facilities and supplies to support caretakers	22.2
	Physician Support	Physicians did not advise caretakers properly on steps to take	14.8
		Poor interactions with physicians, e.g., a “trial and error” method of diagnosis	

* Percentage of caretakers shows the number of interviewed participants that mentioned the specific health literacy factors when asked about their experiences when seeking care.

*Navigation Skills, Understanding of Health Information*, and *Access to Information* also significantly impacted delays in seeking care due to lack of caretaker awareness of the severity or occurrence of the illness or their inability to navigate the complex health system or locate a nearby hospital or clinic, as reflected in the following quotes:

“We did not know the right hospital and health care professionals to handle his case, and this delayed diagnosis.”

“Lack of information about this incident created hours of delay to seeking health care.”

Additionally, caretakers highlighted the lack of *Government, Community, and Physician Support* when seeking care, which influenced the delays in seeking care. Absence of pre-hospital emergency transport and lack of prior information about TBI from physicians led to delays in seeking care and caretakers’ relying on members of the community to seek care on behalf of the patient, as demonstrated in the following quotes:

“Our good friends came for his rescue, and we managed to raise money for a salon car hire…it took us some time to pull together the required sum and caused the delay.”

“The public response to emergencies when such [an] incident happens is still low. Our communities still have ‘I do not care’ tendencies, unless the victim is known to them.”

Caretakers also highlighted the poor access to and *Lack of Medical Resources* in the country and the *Emotional Challenges* that they face, which delay the decision to seek care, as reflected in the quotes:

“After learning about the patient’s accident, I immediately experienced loss of sense [and] confusion…until I composed myself.”

“Family members and friends had to pull money to buy fuel for the ambulance and other expected costs.”

### Factors Influencing Delays to Reaching Care

*Lack of Medical Resources* and *Physician Support* were the two health systems factors that made reaching care the most challenging for caretakers and their patients (Table 2). Caretakers emphasized that the lack of ambulance services and few specialist hospitals in their communities led to delays. They also highlighted limited access to and previous unpleasant encounters with physicians as factors impacting their ability to quickly reach care, as shown in the quotes below:

“The first clinic where we took him could not handle his case. This delayed us to get him [a] fast diagnosis.”

“We moved him from hospital to hospital. We could not get a health professional to diagnose his problem early enough, and we [could not] get him appropriate health care from specialists who would manage his problem.”

**Table 2:** Health literacy themes and sub-themes related to reaching care.

Main Theme	Sub-Theme	Key Examples	% of Caretakers Identifying Sub-Theme*

Extrinsic	Financial Burden	Cost of CT scans, lab tests, and medications	37.0
	Government Support	Subsidized cost of healthcare	25.9
Intrinsic	Emotional Challenges	Feeling anxious due to patient’s condition	0.04
Health Systems	Lack of Medical Resources	Lack of pre-hospital emergency transport	59.2
		Few specialist hospitals	
	Physician Support	Poor communication between physicians and caretakers	22.2
		Physicians urgently attending to patients’ needs	
		The need to save a life first before demanding money	

* Percentage of caretakers shows the number of interviewed participants that mentioned the specific health literacy factors when asked about their experiences when reaching care.

Caretakers also faced emotional burdens, such as dealing with the anxiety and stress of navigating the patient’s condition when trying to reach care. They also underscored the burden that the cost of reaching care and lack of support from the government have on their ability to reach healthcare centers in a timely manner, as shown in the quotes below:

“The Government should work on the traffic jam issue in Kampala and neighboring districts, so as to ease the movement of patients in critical need of fast health care.”

“The Government should provide transport facilities in the hospitals to help with transport.”

### Factors Influencing Delays to Receiving Care

Caretakers identified the lack of medical resources such as CT scanners, beds in the neurosurgery ward, well-equipped pharmacies in MNRH, and other factors such as power outages that make it difficult for patients to receive timely care (Table 3). They also reported the need for governmental support in providing these essential medical resources to help TBI patients receive care when needed, as reflected in the following quotes:

“The Government should have functional machines in place here, instead of doctors telling us to take patients to Nakasero. [They should] also stock medicine in the hospital.”

“The Government should also increase the number of hospitals which handle cases of head injury to reduce congestion in Mulago Hospital and ease service delivery.”

**Table 3:** Health literacy themes and sub-themes related to receiving care.

Main Theme	Sub-Theme	Key Examples	% of Caretakers Identifying Sub-Theme*

Extrinsic	Financial Burdens	Buying medications and paying hospital bills	66.6
		Lack of medication, surgical equipment and scanning machines in hospitals, which requires transfers and referrals	
	Government Support	Need for affordable healthcare	22.2
		Use taxes paid by citizens to provide adequate healthcare	
Intrinsic	Emotional Challenges	Stress from raising money and navigating the healthcare system	11.1
	Physician Support	Putting the patient first before medical bills	11.1
		Prescribing expensive drugs and supplies	
Health System	Lack of Medical Resources	Lack of scanning machines, beds and inconvenient visits to pharmacies to buy drugs	44.4
		Physicians don’t have medical resources to work with	
		Power outages in operating room	

* Percentage of caretakers shows the number of interviewed participants that mentioned the specific health literacy factors when asked about their experiences when receiving care.

Additionally, receiving care presented an immense financial burden on caretakers due to the high cost of referrals, medications, and obtaining scans, as shown in the quotes:

“I had to sell my goats in the farm to raise money to buy medicine after the operation for my patient.”

“We were informed that we need to do a CT scan of the patient’s head…We could not do it fast, because we had to pay for [another] service and we did not have the money at hand by then.”

“We could be told to buy expensive medicine, which is not in the hospital, and at times money is not readily available. It would require us to look for the money, and this would delay treatment.”

Some caretakers highlighted the emotional stress they felt from raising money to attend to their patients’ needs and how this stress was exacerbated by lack of physician support in prescribing cheaper medications, as well as insensitivity to the patients’ needs, as shown in the quotes:

“There was a lot [of] relaxation among the health care personnel to clear his way for operation. I do not know what was happening, but the process was slow, according to my analysis.”

“This might delay her healing process, because when doctors direct you to buy medicine, and you cannot afford, they simply bypass your patient.”

“[Doctors] simply give you an ambulance without fuel, and we had to organize to fuel it to bring the patient here.”

## Discussion

To our knowledge, this is the first qualitative research study evaluating the influence of caretakers’ health literacy on delays to care for TBI patients in an LMIC setting. We elucidated key factors that impede caretakers’ health literacy, thereby contributing to delays to care throughout the care continuum. Importantly, this study revealed that there are more health literacy barriers to seeking care than to reaching and receiving care, suggesting this may be a rate-limiting step in the process of obtaining care.

In order to partake in the decision-making process while seeking care, caretakers must first have access to accurate information about the health issue, where to find help, and who can provide care. Consequently, their ability to successfully assist their patients is dependent upon physician and community support. Caretakers have expressed that their need to have access to health information for TBI patients is unmet due to limited contact with medical personnel. This argument is consistent with other studies that have found that caretakers’ health information needs are sometimes neglected due to poor communication by hospital staff, which can cause delays in seeking and receiving care for patients [17]. There is a need for the healthcare team to spearhead the availability of health information delivery systems for caretakers to ensure they are able to access care-seeking and care management information [18]. Support from medical personnel is also necessary because caretakers depend on them to provide vital health information, such as how to manage the patient’s condition and where to obtain tests, scans, and medications after receiving care.

Lack of support from the community is also highlighted in this study as an important extrinsic factor that contributes to delays in reaching care, especially when caretakers are unable to do so themselves. Caretakers’ health literacy is impacted by their social network, as they will often draw upon or rely on the health literacy skills of others to seek, understand, and use health information [19]. This social support system is important for the caretaker’s emotional well-being and can serve as a stress reliever during the rigorous care reaching process.

Financial burdens were identified as the most significant factor that affects caretaker health literacy in all three delays to care. Uncertainty about the ultimate costs to be incurred can hinder caretakers from seeking or reaching treatment for their patients in the first place. There is, therefore, a need for improved cost-related health literacy that will enlighten caretakers on the potential costs of care in order to make timely decisions on the most cost-effective approach to ultimately receiving care [20]. Furthermore, the government and hospital administrators have a role in ensuring that low-income caretakers are financially supported throughout the process by providing affordable resources and treatments to prevent delays.

After reaching care, TBI patients require urgent medical attention, depending on the severity of injuries. The impact of financial burdens on receiving care manifests as the costs of medications pre- and post-surgery, CT scans, hospital bills, and surgical materials and is exacerbated by the lack of medical resources. A previous study at MNRH found that 5% of TBI patients failed to receive surgery due to infrastructural limitations, 33.6% waited more than four hours to be seen by neurosurgery staff after arrival, and most patients diagnosed with TBI waited for more than a day in the hospital before receiving care due to several financial barriers [21]. The cost of CT scans in Uganda typically ranges from $70 to $132 [22], which can be the average monthly income of a household in Uganda [15]. Catastrophic expenditure is, therefore, likely from imaging alone. A 2010 study by Kushner and colleagues found that district hospitals across many African countries had under-equipped operating rooms and lacked essential medical and surgical supplies [23]. This lack of infrastructure translates to increased financial burden for caretakers and requires them to spend time raising money to cover costs, leading to delays in receiving care. In some instances, caretakers at MNRH are required to pay medical bills, provide certain medical supplies, or undergo tests and scans at other hospitals before physicians can attend to their patients, contributing to delays in receiving care. In order to mitigate such delays, physicians can support caretakers by being more sensitive to and cognizant of these financial challenges and engaging in various strategies to assist caretakers despite these barriers. This could be accomplished through government financial support services or access to good financial advice [7]. There is also a strong need for federal action to provide financial support to caretakers and provide medical resources in public hospitals, especially during the receiving stage of care, to ensure access to quality care for vulnerable populations [24].

Caretakers have expressed that the lack of medical resources within an already complex health system makes reaching and receiving care difficult and exacerbates the impact of financial burdens on caretakers. The most critical resource that increases this delay is the absence of an adequate ambulance system, which forces caretakers to utilize other means of transportation that may be unreliable and increase the risk of poor health outcomes. Patients with transportation barriers also shoulder a greater burden of disease, which may, in part, reflect the relationship between poverty and transportation availability [25]. This relationship is seen in the way the out-of-pocket costs associated with both public and private transportation disproportionately affect low-income caretakers. The amount of time needed to raise money to afford transportation to the hospital was the main reason cited for delays in reaching care. Travel and transportation barriers (e.g., bad roads) lead to poor patient outcomes and increased mortality [25]. About 45% of deaths and 35% of disability-adjusted life-years can be addressed by developing robust emergency care systems in LMICs [26]. Consequently, government support of caretakers can involve strengthening health systems by providing emergency medical resources and cheaper means of reaching care and implementing national programs and policies to improve health literacy [8].

Study findings showed that caretakers’ navigation skills play an important role only during care-seeking but are affected by other health literacy factors identified in this study, such as lack of medical resources and access to and understanding of health information. The ability to look for and make decisions regarding where to receive care and the best means to get there requires adequate navigation skills. Many caretakers reported difficulty in knowing where to find health care facilities—primarily due to lack of knowledge—making them less likely to seek out care. Patients and their caretakers who are able to reach clinics after seeking care often encounter a lack of CT scanners and other resources to address their health needs and are often referred to larger hospitals such as MNRH. This can complicate the care-seeking and reaching processes, contributing to delays in receiving care as patients are moved from one hospital or clinic to another. Caretakers’ ability to maneuver their way through large and complicated health systems directly impacts TBI patient outcomes, especially in time-sensitive situations. Improved access to information regarding the best ways to reach the most appropriate facility will improve caretakers’ self-efficacy and diminish delays in care-seeking.

Notably, the emotional challenges faced by caretakers were the only intrinsic factor highlighted in all three delays to care, which emphasizes how emotionally tasking the process of navigating the healthcare system on behalf of patients can be. This finding could be due to intrinsic factors not being emphasized by the caretakers due to the nature of the questions asked or the tendency to draw attention to extrinsic factors, over which they have no control. The stress of navigating the very complex healthcare system can take a toll on caretakers’ emotional well-being and affect their capacity to process health information. One study posited a social work liaison program, long-term follow-up, and peer support as promising approaches to enhancing coping for families of TBI patients [27].

## Study Limitations

As the interview questions focused on caretakers’ experiences with delays to care, only negative experiences were captured in the responses. Consequently, some participants may have felt their responses reflected poorly on the government, hospital staff, and health system, which might have prevented them from being forthright in their responses. This could also mean that not all of their experiences navigating the healthcare system were captured in their interviews. There was also a lack of perspectives from patients and physicians whose perceptions of care, health literacy, and delays to care will provide a holistic viewpoint regarding factors affecting delays to care in Uganda.

Additionally, this is a single site study at Uganda’s main referral hospital; therefore, study findings are not representative of caretaker experiences navigating healthcare for adult TBI patients throughout the country. This study could be extended to Mbarara Regional Referral Hospital, the only other hospital conducting neurosurgeries for TBI in Uganda. Interviewing caretakers at that location could highlight other caretaker health literacy factors influencing TBI care not captured by participants at MNRH. This limitation could also be mitigated through the continuation of this study with new data from patients and their families. It is important to note that caretakers’ experiences highlighted in this study may or may not reflect experiences surrounding other time-sensitive disease emergencies such as stroke, sepsis, and other forms of trauma, and further research is necessary to elucidate this.

## Conclusion

Several components of caretaker functional health literacy interact with each other and contribute to delays at more than one stage of the care continuum, underscoring the complexity of both the issues underlying delays to care and the potential solutions. However, most of the actionable work needed to reduce delays in care for TBI patients in Uganda falls on extrinsic and health system factors (financial burdens and physician and government support) that influence caretaker functional health literacy. This calls for more emphasis on improving transportation systems, public health education, and access to medical services, particularly for low-income caretakers. Further exploration of how caretakers’ health literacy impacts delays to TBI patient care in LMICs is crucial for the development of interventions targeted at improving their ability to navigate the healthcare system and tailoring health education and communication strategies to their health literacy needs.

Moving forward, healthcare workers and policymakers must pay attention not only to the health education needs of patients but also of their caretakers. Several needs identified in this study, such as improved access to health information and financial and psychosocial assistance, could potentially be addressed with local and government-sponsored programs. Another potential route for impact is federal assistance in improving transportation services throughout the country to overcome barriers that delay care. Caretakers remain at the forefront of healthcare delivery and are important stakeholders in patient care, especially in LMICs. Consequently, allocating funding resources to interventions that support them as they navigate complex health systems will potentially improve patient outcomes.
